# Supplementary material for: The performance model of logistic distribution centers: Quality function deployment based on the Best-Worst Method
Source: PLoS One. 2024 Oct 31;19(10):e0305202. doi: 10.1371/journal.pone.0305202 (PMC11527329; doi:10.1371/journal.pone.0305202)
Supplement: S1 File — (PDF) [file pone.0305202.s001.pdf]

CONSTRUCTS

Respondent 1

	C1	C2	C3	C4	C5
C2	5	1	6	4	4
C5	9	4	4	9	1

Respondent 2

	C1	C2	C3	C4	C5
C5	5	8	3	9	1
C3	4	1	1	8	3

Respondent 3

	C1	C2	C3	C4	C5
C3	6	5	1	7	2
C1	1	9	6	5	1

Respondent 4

	C1	C2	C3	C4	C5
C4	4	1	1	1	2
C5	4	9	9	2	1

Respondent 5

	C1	C2	C3	C4	C5
C5	9	6	6	8	1
C4	5	6	8	1	8

Respondent 6

	C1	C2	C3	C4	C5
C1	1	6	8	1	7
C3	8	3	1	6	1

Respondent 7

	C1	C2	C3	C4	C5
C1	1	2	1	2	8
C5	8	2	3	4	1

Respondent 8

	C1	C2	C3	C4	C5
C5	4	7	6	8	1
C2	3	1	8	3	7

Respondent 9

	C1	C2	C3	C4	C5
C5	1	1	8	3	1
C1	1	4	7	4	1

Respondent 10

	C1	C2	C3	C4	C5
C1	1	4	1	8	7
C5	7	5	5	5	1

Respondent 11

	C1	C2	C3	C4	C5
C5	4	4	6	5	1
C1	1	6	2	8	4

Respondent 12

	C1	C2	C3	C4	C5
C5	3	5	9	9	1
C2	2	1	9	2	5

Respondent 13

	C1	C2	C3	C4	C5
C2	4	1	3	5	2
C5	4	2	8	8	1

Respondent 14

	C1	C2	C3	C4	C5
C1	1	9	5	7	3
C4	7	9	5	1	3

Respondent 15

	C1	C2	C3	C4	C5
C5	4	7	1	7	1
C1	1	7	1	2	4

Respondent 16

	C1	C2	C3	C4	C5
C1	1	8	7	1	3
C2	8	1	1	3	1

Respondent 17

	C1	C2	C3	C4	C5
C5	8	4	9	4	1
C4	5	7	1	1	4

Respondent 18

	C1	C2	C3	C4	C5
C3	4	2	1	8	8
C5	1	7	8	4	1

Respondent 19

	C1	C2	C3	C4	C5
C5	8	6	4	9	1
C5	5	2	5	8	1

Respondent 20

	C1	C2	C3	C4	C5
C5	2	8	6	9	1
C1	1	1	1	8	2

Respondent 21

	C1	C2	C3	C4	C5
C3	9	4	1	9	4
C4	3	7	9	1	5

Respondent 22

	C1	C2	C3	C4	C5

C4	1	8	2	1	4
C2	5	1	6	8	3

Respondent 23

	C1	C2	C3	C4	C5
C1	1	5	5	1	3
C5	3	9	2	1	1

Respondent 24

	C1	C2	C3	C4	C5
C5	4	1	1	2	1
C3	9	2	1	7	1

Respondent 25

	C1	C2	C3	C4	C5
C5	2	5	2	4	1
C4	7	8	6	1	4

Respondent 26

	C1	C2	C3	C4	C5
C5	2	9	1	8	1
C5	6	2	1	3	1

Respondent 27

	C1	C2	C3	C4	C5
C5	2	9	1	6	1
C2	5	1	6	1	9

Respondent 28

	C1	C2	C3	C4	C5
C3	1	9	1	7	1
C2	1	1	9	4	7

Respondent 29

	C1	C2	C3	C4	C5
C5	6	4	9	9	1

C2	3	1	1	3	4

Respondent 30

	C1	C2	C3	C4	C5
C2	8	9	5	9	1
C5	8	1	5	9	1

Respondent 31

	C1	C2	C3	C4	C5
C5	8	6	6	7	1
C3	3	9	1	4	6

Respondent 32

	C1	C2	C3	C4	C5
C5	8	1	6	9	1
C2	9	1	8	4	1

Respondent 33

	C1	C2	C3	C4	C5
C3	9	6	1	4	2
C1	1	4	9	7	8

Respondent 34

	C1	C2	C3	C4	C5
C5	9	1	1	7	1
C3	2	4	1	4	1

Respondent 35

	C1	C2	C3	C4	C5
C1	1	1	1	9	3
C2	1	1	7	4	3

Respondent 36

	C1	C2	C3	C4	C5
C5	6	1	9	6	1
C1	1	9	2	8	6

Respondent 37

	C1	C2	C3	C4	C5
C5	2	7	5	1	1
C1	1	5	1	7	2

The TB contruct

Respondent 1

	C1	C2	C3	C4
C3	5	9	1	9
C2	6	1	9	4

Respondent 2

	C1	C2	C3	C4
C4	1	4	6	1
C1	1	3	9	1

Respondent 3

	C1	C2	C3	C4
C4	8	6	6	1
C2	8	1	3	6

Respondent 4

	C1	C2	C3	C4
C4	9	5	8	1
C1	1	5	6	9

Respondent 5

	C1	C2	C3	C4
C2	8	1	5	6
C4	4	6	7	1

Respondent 6

	C1	C2	C3	C4
C1	1	4	1	3
C4	3	8	9	1

Respondent 7

	C1	C2	C3	C4
C1	1	9	7	1
C4	1	1	8	1

Respondent 8

	C1	C2	C3	C4
C3	3	5	1	2
C1	1	3	3	6

Respondent 9

	C1	C2	C3	C4
C1	1	1	9	8
C3	9	9	1	3

Respondent 10

	C1	C2	C3	C4
C4	2	5	8	1
C2	4	1	3	5

Respondent 11

	C1	C2	C3	C4
C4	2	3	6	1
C1	1	9	7	2

Respondent 12

	C1	C2	C3	C4
C3	1	9	1	2
C4	1	4	2	1

Respondent 13

	C1	C2	C3	C4
C4	6	7	9	1
C3	1	2	1	9

Respondent 14

	C1	C2	C3	C4
C2	7	1	8	2
C4	1	2	4	1

Respondent 15

	C1	C2	C3	C4
C3	9	2	1	6
C4	2	7	6	1

Respondent 16

	C1	C2	C3	C4
C1	1	4	3	4
C4	4	3	2	1

Respondent 17

	C1	C2	C3	C4
C1	1	7	1	5
C2	7	1	5	3

Respondent 18

	C1	C2	C3	C4
C4	3	7	2	1
C1	1	2	3	3

Respondent 19

	C1	C2	C3	C4
C4	8	6	8	1
C1	1	9	1	8

Respondent 20

	C1	C2	C3	C4
C4	1	7	7	1
C1	1	3	2	1

Respondent 21

	C1	C2	C3	C4
C4	5	7	1	1
C1	1	4	4	5

Respondent 22

	C1	C2	C3	C4

C4	4	9	9	1
C2	7	1	8	9

Respondent 23

	C1	C2	C3	C4
C1	1	4	3	2
C4	2	9	3	1

Respondent 24

	C1	C2	C3	C4
C4	9	5	5	1
C1	1	1	7	9

Respondent 25

	C1	C2	C3	C4
C4	1	8	8	1
C2	6	1	7	8

Respondent 26

	C1	C2	C3	C4
C1	1	6	4	5
C3	4	1	1	4

Respondent 27

	C1	C2	C3	C4
C1	1	4	5	3
C4	3	3	7	1

Respondent 28

	C1	C2	C3	C4
C4	9	8	5	1
C1	1	6	6	9

Respondent 29

	C1	C2	C3	C4
C4	8	1	2	1

C2	3	1	6	1

Respondent 30

	C1	C2	C3	C4
C2	6	1	5	9
C3	2	5	1	7

Respondent 31

	C1	C2	C3	C4
C2	7	1	5	3
C1	1	7	3	8

Respondent 32

	C1	C2	C3	C4
C1	1	7	9	2
C3	9	7	1	9

Respondent 33

	C1	C2	C3	C4
C4	5	2	5	1
C3	7	5	1	5

Respondent 34

	C1	C2	C3	C4
C4	1	6	6	1
C1	1	7	8	1

Respondent 35

	C1	C2	C3	C4
C3	6	3	1	3
C1	1	7	6	4

Respondent 36

	C1	C2	C3	C4
C4	8	3	5	1
C3	8	5	1	5

Respondent 37

	C1	C2	C3	C4
C1	1	3	7	3
C4	3	8	5	1

The RB construct

Respondent 1

	C1	C2	C3	C4
C2	9	1	2	1
C1	1	9	3	1

Respondent 2

	C1	C2	C3	C4
C2	9	1	3	7
C4	5	7	5	1

Respondent 3

	C1	C2	C3	C4
C1	1	7	2	9
C4	9	6	5	1

Respondent 4

	C1	C2	C3	C4
C3	3	5	1	9
C4	6	3	9	1

Respondent 5

	C1	C2	C3	C4
C4	1	2	2	1
C2	5	1	6	2

Respondent 6

	C1	C2	C3	C4
C1	1	7	8	2
C4	2	3	6	1

Respondent 7

	C1	C2	C3	C4
C1	1	3	8	9
C4	9	3	7	1

Respondent 8

	C1	C2	C3	C4
C4	7	2	8	1
C1	1	7	1	7

Respondent 9

	C1	C2	C3	C4
C2	7	1	4	4
C4	6	4	4	1

Respondent 10

	C1	C2	C3	C4
C4	9	5	4	1
C2	7	1	6	5

Respondent 11

	C1	C2	C3	C4
C4	8	5	3	1
C2	7	1	4	5

Respondent 12

	C1	C2	C3	C4
C3	5	9	1	8
C1	1	7	5	5

Respondent 13

	C1	C2	C3	C4
C2	6	1	1	6
C4	2	6	4	1

Respondent 14

	C1	C2	C3	C4
C4	1	8	2	1
C2	6	1	6	8

Respondent 15

	C1	C2	C3	C4
C4	6	8	5	1
C3	6	5	1	5

Respondent 16

	C1	C2	C3	C4
C2	6	1	8	1
C1	1	6	6	2

Respondent 17

	C1	C2	C3	C4
C4	2	4	6	1
C1	1	2	1	2

Respondent 18

	C1	C2	C3	C4
C4	7	2	2	1
C2	6	1	5	2

Respondent 19

	C1	C2	C3	C4
C4	5	4	6	1
C2	8	1	4	4

Respondent 20

	C1	C2	C3	C4
C1	1	3	3	5
C4	5	6	8	1

Respondent 21

	C1	C2	C3	C4
C2	3	1	5	6
C1	1	3	9	3

Respondent 22

	C1	C2	C3	C4

C1	1	7	4	9
C4	9	9	3	1

Respondent 23

	C1	C2	C3	C4
C2	3	1	1	1
C4	9	1	5	1

Respondent 24

	C1	C2	C3	C4
C3	1	2	1	6
C4	2	5	6	1

Respondent 25

	C1	C2	C3	C4
C2	9	1	1	7
C3	3	1	1	1

Respondent 26

	C1	C2	C3	C4
C4	2	5	8	1
C2	3	1	6	5

Respondent 27

	C1	C2	C3	C4
C1	1	7	9	7
C4	7	9	4	1

Respondent 28

	C1	C2	C3	C4
C3	4	4	1	6
C1	1	7	4	2

Respondent 29

	C1	C2	C3	C4
C4	7	1	8	1

C3	1	4	1	8

Respondent 30

	C1	C2	C3	C4
C4	6	1	6	1
C1	1	1	4	6

Respondent 31

	C1	C2	C3	C4
C1	1	4	4	7
C4	7	1	7	1

Respondent 32

	C1	C2	C3	C4
C4	7	4	4	1
C2	1	1	5	4

Respondent 33

	C1	C2	C3	C4
C4	8	7	4	1
C4	7	4	6	1

Respondent 34

	C1	C2	C3	C4
C2	9	1	5	9
C4	6	9	4	1

Respondent 35

	C1	C2	C3	C4
C3	7	2	1	7
C4	6	9	7	1

Respondent 36

	C1	C2	C3	C4
C4	3	7	5	1
C2	3	1	8	7

Respondent 37

	C1	C2	C3	C4
C1	1	1	9	9
C4	9	3	7	1

The RS construct

Respondent 1

	C1	C2	C3	C4
C1	1	8	5	4
C4	4	3	7	1

Respondent 2

	C1	C2	C3	C4
C4	5	1	3	1
C1	1	3	8	5

Respondent 3

	C1	C2	C3	C4
C4	9	7	4	1
C1	1	7	1	9

Respondent 4

	C1	C2	C3	C4
C3	2	7	1	5
C1	1	7	2	3

Respondent 5

	C1	C2	C3	C4
C4	5	1	3	1
C3	8	7	1	3

Respondent 6

	C1	C2	C3	C4
C4	4	9	1	1
C2	5	1	3	9

Respondent 7

	C1	C2	C3	C4
C3	1	5	1	9
C2	6	1	5	9

Respondent 8

	C1	C2	C3	C4
C2	8	1	6	1
C1	1	8	4	4

Respondent 9

	C1	C2	C3	C4
C4	7	2	2	1
C1	1	5	6	7

Respondent 10

	C1	C2	C3	C4
C2	7	1	6	4
C4	2	4	3	1

Respondent 11

	C1	C2	C3	C4
C4	2	2	7	1
C2	5	1	2	2

Respondent 12

	C1	C2	C3	C4
C1	1	1	7	9
C3	7	9	1	7

Respondent 13

	C1	C2	C3	C4
C4	8	9	9	1
C2	3	1	4	9

Respondent 14

	C1	C2	C3	C4
C4	1	3	8	1
C3	9	1	1	8

Respondent 15

	C1	C2	C3	C4
C4	6	5	4	1
C3	7	2	1	4

Respondent 16

	C1	C2	C3	C4
C2	4	1	3	7
C1	1	4	3	8

Respondent 17

	C1	C2	C3	C4
C4	4	8	3	1
C1	1	4	1	4

Respondent 18

	C1	C2	C3	C4
C1	1	6	6	5
C4	5	8	7	1

Respondent 19

	C1	C2	C3	C4
C4	4	1	1	1
C2	9	1	5	1

Respondent 20

	C1	C2	C3	C4
C4	3	7	8	1
C2	4	1	4	7

Respondent 21

	C1	C2	C3	C4
C2	4	1	7	4
C4	4	4	4	1

Respondent 22

	C1	C2	C3	C4

C4	5	6	8	1
C2	4	1	9	6

Respondent 23

	C1	C2	C3	C4
C1	1	6	7	5
C3	7	1	1	9

Respondent 24

	C1	C2	C3	C4
C4	1	7	9	1
C1	1	1	6	1

Respondent 25

	C1	C2	C3	C4
C4	3	7	6	1
C2	2	1	9	7

Respondent 26

	C1	C2	C3	C4
C3	1	1	1	2
C2	1	1	1	7

Respondent 27

	C1	C2	C3	C4
C4	3	7	6	1
C2	1	1	2	7

Respondent 28

	C1	C2	C3	C4
C1	1	4	7	9
C2	4	1	8	7

Respondent 29

	C1	C2	C3	C4
C1	1	3	5	3

C4	3	1	3	1

Respondent 30

	C1	C2	C3	C4
C4	7	5	4	1
C1	1	1	4	7

Respondent 31

	C1	C2	C3	C4
C4	3	4	2	1
C1	1	6	6	3

Respondent 32

	C1	C2	C3	C4
C1	1	4	9	1
C4	1	1	3	1

Respondent 33

	C1	C2	C3	C4
C1	1	1	1	1
C3	1	4	1	5

Respondent 34

	C1	C2	C3	C4
C2	1	1	3	6
C4	6	6	5	1

Respondent 35

	C1	C2	C3	C4
C4	7	1	6	1
C1	1	9	5	7

Respondent 36

	C1	C2	C3	C4
C3	5	1	1	4
C1	1	6	5	7

Respondent 37

	C1	C2	C3	C4
C4	1	4	3	1
C1	1	6	7	1

The EB construct

Respondent 1

	C1	C2	C3	C4
C4	2	5	2	1
C1	1	6	3	2

Respondent 2

	C1	C2	C3	C4
C1	1	8	2	9
C2	8	1	4	5

Respondent 3

	C1	C2	C3	C4
C4	1	4	9	1
C3	2	4	1	9

Respondent 4

	C1	C2	C3	C4
C3	8	2	1	2
C1	1	6	8	6

Respondent 5

	C1	C2	C3	C4
C1	1	3	4	1
C4	1	5	3	1

Respondent 6

	C1	C2	C3	C4
C4	7	7	2	1
C1	1	1	3	7

Respondent 7

	C1	C2	C3	C4
C2	3	1	6	3
C4	9	3	1	1

Respondent 8

	C1	C2	C3	C4
C4	6	7	8	1
C1	1	4	9	6

Respondent 9

	C1	C2	C3	C4
C4	1	6	9	1
C1	1	1	4	1

Respondent 10

	C1	C2	C3	C4
C3	8	7	1	6
C2	8	1	7	4

Respondent 11

	C1	C2	C3	C4
C1	1	3	1	2
C2	3	1	1	6

Respondent 12

	C1	C2	C3	C4
C4	6	7	1	1
C3	6	5	1	1

Respondent 13

	C1	C2	C3	C4
C1	1	5	2	2
C4	2	9	6	1

Respondent 14

	C1	C2	C3	C4
C2	6	1	1	3
C4	7	3	8	1

Respondent 15

	C1	C2	C3	C4
C4	4	5	3	1
C2	8	1	8	5

Respondent 16

	C1	C2	C3	C4
C1	1	5	4	7
C4	7	2	5	1

Respondent 17

	C1	C2	C3	C4
C4	3	2	2	1
C2	4	1	2	2

Respondent 18

	C1	C2	C3	C4
C4	9	4	7	1
C2	7	1	7	4

Respondent 19

	C1	C2	C3	C4
C4	1	6	4	1
C2	6	1	8	6

Respondent 20

	C1	C2	C3	C4
C2	4	1	8	2
C4	4	2	3	1

Respondent 21

	C1	C2	C3	C4
C4	5	1	2	1
C2	6	1	9	1

Respondent 22

	C1	C2	C3	C4

C4	8	4	6	1
C3	1	6	1	6

Respondent 23

	C1	C2	C3	C4
C4	9	9	3	1
C2	9	1	4	9

Respondent 24

	C1	C2	C3	C4
C2	6	1	3	3
C4	8	3	2	1

Respondent 25

	C1	C2	C3	C4
C2	1	1	7	9
C4	6	9	8	1

Respondent 26

	C1	C2	C3	C4
C4	9	4	9	1
C3	1	8	1	9

Respondent 27

	C1	C2	C3	C4
C4	2	9	7	1
C2	9	1	4	9

Respondent 28

	C1	C2	C3	C4
C4	8	1	2	1
C2	6	1	5	1

Respondent 29

	C1	C2	C3	C4
C2	4	1	9	3

C4	5	3	7	1

Respondent 30

	C1	C2	C3	C4
C4	7	9	6	1
C2	2	1	5	9

Respondent 31

	C1	C2	C3	C4
C2	9	1	2	5
C1	1	9	5	7

Respondent 32

	C1	C2	C3	C4
C4	3	2	6	1
C3	4	7	1	6

Respondent 33

	C1	C2	C3	C4
C4	8	3	4	1
C2	8	1	9	3

Respondent 34

	C1	C2	C3	C4
C3	7	1	1	6
C1	1	1	7	7

Respondent 35

	C1	C2	C3	C4
C1	1	8	2	6
C4	6	4	9	1

Respondent 36

	C1	C2	C3	C4
C1	1	3	5	3
C2	3	1	3	1

Respondent 37

	C1	C2	C3	C4
C1	1	9	5	2
C2	9	1	5	7

the AS construct

Respondent 1

	C1	C2	C3	C4
C2	7	1	3	9
C4	3	9	1	1

Respondent 2

	C1	C2	C3	C4
C4	9	9	8	1
C3	7	8	1	8

Respondent 3

	C1	C2	C3	C4
C4	5	9	7	1
C3	6	9	1	7

Respondent 4

	C1	C2	C3	C4
C4	4	4	2	1
C3	6	3	1	2

Respondent 5

	C1	C2	C3	C4
C3	7	6	1	3
C4	3	8	3	1

Respondent 6

	C1	C2	C3	C4
C1	1	8	9	5
C4	5	5	8	1

Respondent 7

	C1	C2	C3	C4
C4	6	9	4	1
C1	1	6	4	6

Respondent 8

	C1	C2	C3	C4
C4	3	3	3	1
C1	1	7	1	3

Respondent 9

	C1	C2	C3	C4
C3	2	3	1	1
C2	2	1	3	9

Respondent 10

	C1	C2	C3	C4
C3	2	3	1	8
C4	4	3	8	1

Respondent 11

	C1	C2	C3	C4
C2	1	1	8	1
C4	6	1	3	1

Respondent 12

	C1	C2	C3	C4
C4	1	4	6	1
C3	6	1	1	6

Respondent 13

	C1	C2	C3	C4
C2	1	1	8	6
C4	8	6	6	1

Respondent 14

	C1	C2	C3	C4
C3	7	7	1	8
C1	1	2	7	3

Respondent 15

	C1	C2	C3	C4
C3	1	6	1	4
C4	5	3	4	1

Respondent 16

	C1	C2	C3	C4
C4	6	3	9	1
C1	1	7	5	6

Respondent 17

	C1	C2	C3	C4
C1	1	3	6	7
C4	7	3	8	1

Respondent 18

	C1	C2	C3	C4
C4	1	1	3	1
C2	1	1	6	1

Respondent 19

	C1	C2	C3	C4
C4	7	8	5	1
C2	5	1	6	8

Respondent 20

	C1	C2	C3	C4
C1	1	7	1	2
C4	2	2	2	1

Respondent 21

	C1	C2	C3	C4
C4	9	1	6	1
C1	1	2	4	9

Respondent 22

	C1	C2	C3	C4

C1	1	6	2	8
C2	6	1	7	2

Respondent 23

	C1	C2	C3	C4
C1	1	4	7	1
C4	1	5	4	1

Respondent 24

	C1	C2	C3	C4
C2	5	1	3	5
C4	7	5	5	1

Respondent 25

	C1	C2	C3	C4
C3	8	3	1	8
C4	9	7	8	1

Respondent 26

	C1	C2	C3	C4
C1	1	3	9	1
C2	3	1	7	9

Respondent 27

	C1	C2	C3	C4
C2	1	1	5	6
C4	9	6	7	1

Respondent 28

	C1	C2	C3	C4
C2	4	1	6	1
C1	1	4	5	1

Respondent 29

	C1	C2	C3	C4
C2	7	1	4	3

C3	5	4	1	1

Respondent 30

	C1	C2	C3	C4
C4	5	8	7	1
C2	3	1	4	8

Respondent 31

	C1	C2	C3	C4
C4	2	9	6	1
C1	1	1	7	2

Respondent 32

	C1	C2	C3	C4
C3	9	4	1	5
C2	1	1	4	6

Respondent 33

	C1	C2	C3	C4
C3	3	1	1	4
C2	9	1	1	1

Respondent 34

	C1	C2	C3	C4
C2	5	1	8	8
C3	7	8	1	7

Respondent 35

	C1	C2	C3	C4
C1	1	4	6	7
C4	7	4	3	1

Respondent 36

	C1	C2	C3	C4
C1	1	1	7	8
C2	1	1	3	3

Respondent 37

	C1	C2	C3	C4
C2	3	1	9	7
C4	2	7	7	1
