# Supplementary material for: The performance model of logistic distribution centers: Quality function deployment based on the Best-Worst Method
Source: PLoS One. 2024 Oct 31;19(10):e0305202. doi: 10.1371/journal.pone.0305202 (PMC11527329; doi:10.1371/journal.pone.0305202)
Supplement: Attachment — Submitted filename: PONE-D-23-31891.Responses to reviewers.pdf [file pone.0305202.s003.pdf]

Dear Academic Editor and Reviewers,

I hope this letter finds you well. I am writing to submit the revised version of my manuscript, "The performance model of logistic distribution centers: Quality Function Deployment based on the Best-Worst Method," which was originally submitted on October 1st 2023. I appreciate the careful review of the manuscript by the reviewers and the valuable feedback provided. I would like to express my gratitude for the constructive comments and suggestions made by the reviewers. Their insights have significantly contributed to the improvement of the manuscript. In response to the reviewers' comments, we have made the following key revisions:

Responses to Reviewer #1:

The paper aims to evaluate the performance of logistic distribution centers (LDCs) by Quality Function Deployment (QFD) based on the Best-Worst Method (BWM). Some critical concerns are mentioned below.

Comment# 1. In abstract, we cannot recognize the novelties and contributions of the paper.

Answer: Thanks so much for your comments. We already revised the abstract to present some originalities of the article (**Seeing Rows 23-30 in red texts**).

Comment# 2. What are the research gaps in the literature?

Answer: Thanks so much for your comments. We already revised the literature review section to present some research gaps (**Seeing Rows 180-193 in red texts**).

Comment# 3. What are your arguments to prefer BWM instead of other weighting methods, such as AHP, FUCOM, LMAW, LBWA, etc. Discuss BWM's advantages over other methods mentioned.

Answer: Thanks so much for your comments. We already revised the introduction section to present why BWM is used in our paper. Besides, BWM's advantages over other methods are discussed in this section (**Seeing Rows 59-74 in red texts**).

Comment# 4. Another deficiency of the paper is the BWM literature. You should add a new sub-section in LR about recent BWM papers in PLOS ONE and other respectful journals. For instance, Exploring factors affecting airport selection during the COVID-19 pandemic from air

cargo carriers' perspective through the triangular fuzzy Dombi-Bonferroni BWM methodology; Assessment and risk mitigation on halal meat supply chain using fuzzy best-worst method (BWM) and risk mitigation number (RMN); The Suitability-Feasibility-Acceptability Strategy Integrated with Bayesian BWM-MARCOS Methods to Determine the Optimal Lithium Battery Plant Located in South America; Neighborhood selection for a newcomer via a novel BWM-based revised MAIRCA integrated model: a case from the Coquimbo-La Serena conurbation, Chile; Prioritization of land consolidation projects using the multi-criteria Best-Worst Method: a case study from Poland.

Answer: Thanks so much for your comments. We already inserted one sub-section "2.2. Best-Worst method" to provide an overview of BWM. Besides, your suggested studies are valuable sources of reference in this sub-section (**Seeing Rows 111-136 in red texts**).

Comment# 5. Check the whole file regarding English language and fix some grammatical mistakes.

Answer: Thanks so much for your comments. We had one English native speaker review the manuscript and correct any grammatical errors.

Responses to Reviewer #2:

The authors of this paper conducted a study on the performance evaluation of LDCs by integrating the BWM and QFD methods. The overall structure and content of this work are acceptable. However, there are some comments and suggestions for the authors to consider when improving the quality of your work. Please see the attachment for details of my comments.

The authors of this paper conducted a study on the performance evaluation of LDCs by integrating the BWM and QFD methods. The overall structure and content of this work are acceptable. However, the following suggestions are provided for the authors to consider when improving the quality of your work:

Comment# 1. The research background of the paper does not clearly explain why the QFD and BWM methods are necessary and how they are relevant to the research problem. Where do these methods align with the study?

Answer: Thanks so much for your comments. We already revised the introduction section to explain why the QFD and BWM methods are necessary in our study and how they are relevant to the evaluation of LDCs' performance (**Seeing Rows 46-74 in green and red texts**). Moreover, we present where these methods align with the study in the section "3.1. Research framework" (**Seeing Rows 196-205 in green texts**).

Comment# 2. The literature review section needs to be rewritten. Currently, it is simply a list without explaining the connections between the literature and why these specific ones were chosen. As we all know there is a vast amount of research on the performance evaluation of LDCs, so we recommend the authors present and compare the literature in a table format. Additionally, we did not see any analysis of the existing literature, particularly the shortcomings of previous studies and how this paper intends to address them.

Answer: Thanks so much for your comments. We already revised the literature section to address your concerns. More specifically, we inserted one section to discuss the advantages and challenges of adopting BWM (**Seeing Rows 111-136 in red texts**). Additionally, we already inserted two paragraphs in the literature section to present some research gaps that were fulfilled

in this manuscript (**Seeing Rows 180-192 in red texts**). Further, we presented and compared the previous literature in a table format (**Seeing Table 1 in Row 571 in green texts**).

Comment# 3. What is the logical framework of the CRA system constructed in this paper? It appears that the authors used the SERVQUAL model, but what is the relationship or difference between it and the existing evaluation indicators?

Answer: Thanks so much for your comments. To suggest strategies to improve LDCs' performance, this paper started with identifying customer requirements (i.e., CRAs) on the basis of the SERQUAL model and the operating features of LDCs. It has been argued that the SERQUAL model is exceedingly useful in figuring out customer requirements in the service industry, including five primary constructs (i.e., Tangibility, Reliability, Responsiveness, Empathy, and Assurance). Yet, these constructs' observed items are referred to the relevant literature regarding LDCs.

Comment# 4. The methods used in the paper are mature methods proposed by others, and we are concerned about the originality or novelty of the paper itself. The authors should summarize and emphasize the innovative aspects of this study.

Answer: Thanks so much for your comments. the originality or novelty of the paper is summarized in the conclusion section (**Seeing Rows 415-434 in green texts**). On top of that, we already revised the abstract to present some originalities of the article (Seeing Rows 23-30 in red texts).

Comment# 5. The paper does not provide a clear description of how to transition from CRAs to STRs. It seems that STRs were derived based on expert opinions without utilizing the HOQ tool.

Answer: Thanks so much for your comments. We would like to explain how to translate from CRAs to STRs, as follows:

First of all, we explored CRAs from the literature review and the operating features of LDCs. Then, BWM was used to measure CRAs' relative weight with the sample size of 21 respondents. Afterwards, we identified STRs from LDCs' standard operating procedures (SOPs)

and experts' consultations (as shown in Figure 3 of the manuscript). Next, to set up HoQ for translating CRAs into STRs, the paper calculated the central relationship matrix and the correlation matrix by surveying 7 experts. After that, the weight of STRs was determined by Formulas (5) – (10). Finally, we ranked STRs' relative weight to prioritize the resource allocation.

Comment# 6. The case in the paper involves a multi-person collaborative decision-making problem. We recommend the author to use group decision-making methods, especially considering the influence of different experts' weights on the decision outcome.

Answer: Thanks so much for your comments. We really appreciate your suggestion. Yet, the locations of LDCs for the empirical case are so far. Accordingly, it will not be easy for us to run a roundtable discussion for group decision-making. Thus, we declared this point as a research limitation in our manuscript to accept your criticism (**Seeing Rows 439-442 in green texts**).

Comment# 7. The weights of the indicators have a significant impact on the final ranking of LDCs. The paper uses subjective evaluation data for this, so to enhance the robustness of the research, we suggest the author perform sensitivity analysis on the indicator weights. The following papers related to QFD and MCDMs may provide insights into this issue and should be referenced. (a) A Hesitant Fuzzy Linguistic QFD Approach for Formulating Sustainable Supplier Development Programs doi:10.1016/j.ijpe.2022.108428; (b) Large-scale group decision-making for prioritizing engineering characteristics in quality function deployment under comparative linguistic environment. doi:10.1016/j.asoc.2022.109359, (c) Enhancing Quality Function Deployment Through the Integration of Rough Set and Ordinal Priority Approach: A Case Study in Electric Vehicle Manufacturing etc.

Answer: Thanks so much for your comments. We already inserted one section to present a sensitive analysis (Seeing Rows 371-384 in green and red texts).

Comment# 8. The paper lacks a comparison with other quantitative research methods, which prevents it from demonstrating the superiority of this study. The case study only proves the feasibility of the research methods used in this paper.

Answer: Thanks so much for your comments. We already inserted one section to compare STRs' ranking from the different QFD models (**Seeing Rows 385-404 in green and red texts**).

Comment# 9. The paper contains numerous grammar and spelling mistakes. For example, the LCD in section 2.2 should be LDC and the MWM in section 3.1 should be BWM. We recommend the author have a native English speaker carefully review and improve the writing.

Answer: Thanks so much for your comments. We had one English native speaker review the manuscript and correct any grammatical errors.

Responses to Reviewer #3: Thank you for this contribution. This is an interesting and timely manuscript. A good piece of work has been done, and I recommend its acceptance. The authors need to update the literature, especially in recent years.

Please review the below references:

Elhegazy, H., Ebid, A., Mahdi, I., Haggag, S., & Abdul-Rashied, I. (2021). Implementing QFD in decision making for selecting the optimal structural system for buildings. Construction Innovation, 21(2), 345-360. doi:10.1108/CI-12-2019-0149

Elhegazy, H., Mahmoud, W., Eid, M., & Khairy, N. (2022). Quality Function Deployment Framework for Selecting Optimal Greenhouse Microclimate Control System. Journal of Industrial Integration and Management. doi:10.1142/S2424862222500105.

Answer: Thanks so much for your comments. We revised the manuscript by incorporating some suggested research (**Seeing Rows 46-58 in green texts**).

Responses to Academic Editor: Thanks so much for your suggestion. We already deleted Figure 3 from our manuscripts.

Once again, thank you for your dedication to advancing scientific knowledge and for your commitment to maintaining the high standards of PLOS ONE. We greatly appreciate your support throughout this process. If you have any further questions or require additional information, please do not hesitate to contact us.

Sincerely,

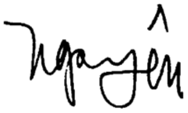

Nguyen Tan Huynh

huynhtannguyen@dntu.edu.vn

Faculty of Economics-Management, Dong Nai Technology University, Bien Hoa City, Vietnam.
