# Supplementary material for: The performance model of logistic distribution centers: Quality function deployment based on the Best-Worst Method
Source: PLoS One. 2024 Oct 31;19(10):e0305202. doi: 10.1371/journal.pone.0305202 (PMC11527329; doi:10.1371/journal.pone.0305202)
Supplement: Attachment — Submitted filename: R2.PONE-D-23-31891.Responses to reviewers.pdf [file pone.0305202.s004.pdf]

Dear Academic Editor and Reviewers,

I hope this letter finds you well. I am writing to submit the revised version of my manuscript, "The performance model of logistic distribution centers: Quality Function Deployment based on the Best-Worst Method," which was originally submitted on October 1st 2023. I appreciate the careful review of the manuscript by the reviewers and the valuable feedback provided. I would like to express my gratitude for the constructive comments and suggestions made by the reviewers. Their insights have significantly contributed to the improvement of the manuscript. In response to the reviewers' comments, we have made the following key revisions. Particulary, the red texts are responses to Reviewers.

Responses to Reviewer #4:

Thanks for the opportunity to review the paper. The reviews made are sufficient for the paper publication. I recommend the publication of the article.

Answer: Thanks so much for your suggestion. We are very grateful to your effort for reviewing our manuscript.

Responses to Reviewer #5: Although some work has been done in this article, there are still some issues that need improvement:

(1) The research motivation of this article is not clear enough, which is why the study was conducted and why BWM was used. From my understanding, it should be an improved version of AHP. Its characteristics should be emphasized and clearly described.

Answer: Thanks so much for your comment. We are very grateful to your effort for reviewing our manuscript. We would like to explain as follows:

This study was carried out from three main research motivations:

The first one is the indispensable role of Logistical Distribution Centers (LDCs) to the economic growth of nations. Accordingly, improving LDCs' performance has been receiving much attention from academics and practitioners. Still, there is little research on how to improve LDCs' performance. **The first motivation is stated in Rows 40-49 in our manuscript**.

The second one is that the pertinent research in terms of boosting LDCs' operating performance often relates to customer perspectives, which can be defined as "what" issues. However, the strategies to satisfy customer needs are closely associated with service technical requirements (STRs) of LDCs, which can be defined as "how" issues. More crucially, the way to translating customer needs (i.e., CRAs) into specific engineering and design requirements (i.e., STRs) is still questionable in the relevant literature. **This motivation is stated in Rows 50-62**.

And the last motivation is usage of Best-Worst Method (BWM) to overcome the drawback of pairwise comparison-based methods, for instance, Analytic Hierarchy Process (AHP), Analytic network process (ANP). It is argued that BWM requires fewer comparison pairs than other pairwise comparison methods (i.e., AHP, ANP). For instance, BWM only needs $(2n-3)$ pairwise comparisons, while AHP necessitates $n(n-1)/2$ ones. Besides, BWM uses integers from 1, 2,…, and 9 to do pairwise comparisons while AHP and ANP require both integer numbers and fractional numbers. Accordingly, BWM can be easily applied in many real-world cases in terms of evaluating and prioritizing different options based on MCDA. **This motivation is stated in Rows 63-78**.

On top of that the characteristics of BWM were emphasized and clearly described in **Rows 116-142**.

(2) The contribution of this article needs to be clarified, especially in terms of theoretical research. The current form only emphasizes the applicability of BWM. So is there any theoretical contribution?

Answer: Thanks so much for your comment. We are very grateful to your effort for reviewing our manuscript. Some theoretical contributions of this paper are as follows:

First, the adoption of the SERQUAL model allows to explore and identify CRAs' of the LDCs' operations, which include 20 CRAs and 5 dimensions. This is also the foundation for LDCs' operators in building service management requirements to satisfy their customers' requirements. It is illustrated that this model can be extended to further investigate CRAs for a specific operation of LDCs' performance, such as customer services, or business affairs.

Second, the utilization of the the QFD (Quality Function Deployment) model helps decision-makers in translating customer needs (i.e., CRAs) into specific engineering and design requirements (i.e., STRs). Although QFD is not new, this is the first time the "how" issues in the

LDCs can be solved by the implementation of QFD. It would be said that the QDF model can be applicable in the design process and product development of various distribution systems, for instance, electric power distribution, gas distribution systems, agricultural product distribution, etc.

The last theoretical contribution is to adopt BWM to estimate the vector of priority weight of CRAs and STRs. To the best of our knowledge, this is also the first time BWM is studied to assess operations of LDCs. Thus, thanks to some strengths of BWM, as mentioned earlier, it can be efficiently used for multiple-criteria decision analysis (MCDA).

By the way, the theoretical and practical contributions of this paper are summarized in the conclusion section (**Seeing Rows 442-447**).

(3) The literature review in this article lacks logic and completeness, for example, some of the latest relevant literature is missing, such as research literature on BWM, DOI: 10.1016/j-eswa.2023.121227, the authors can update it.

Answer: Thanks so much for your suggestion. The suggested research is really informative. So, we referred to this paper for specific details of BWM (**Seeing Rows 131-132**). Besides, as requested by the another reviewer, the paper also updates some latest research on BWM and QFD (**Seeing Rows 94, 101, 129**).

(4) The conclusion of this article needs to be reorganized, clarifying the theoretical and applied contributions, as well as future research prospects.

Answer: Thanks so much for your suggestion. The conclusion of this article was reorganized to reflect the theoretical and applied contributions (**Seeing Rows 442-447**. And the future research prospects was illustrated in the last part of the paper (**Seeing Rows 448-455**).

(5) The format and language of this article need to be carefully revised.

Answer: Thanks so much for your suggestion. We had one English native speaker revise our manuscript to ensure that it is grammatical.

Responses to Reviewer #6:

(1) Literature review part should be extended. There are many important researches on QFD and BWM, the authors should consider these studies.

Answer: Thanks so much for your suggestion. We revised the literature review part by updating some latest research on QFD and BWM (**Seeing Rows 131-132; Rows 94, 101, 129**).

(2) Managerial implications and discussions should be provided.

Answer: Thanks so much for your suggestion. The managerial implications and discussions are shown in Section 4 (**Seeing Rows 331-377**)

(3) The authors should explain how they obtained the data, who are the experts, what are their backgrounds.

Answer: Thanks so much for your suggestion. We would like to explain as follows:

a) The way to collect data:

According to the Vietnam Ministry of Industry and Trade (2023), there are 33 LDCs across Vietnam as of 2022. More specifically, the vast majority of these are located in the major cities and industrial provinces of Vietnam, such as HCM city, Hanoi, Danang, Dong Nai, Binh Duong, and Hai Phong. Some LDCs with a major operational scale include Gemadept, TBS Logistics, Vinalines Logistics, Saigon Newport, Transimex, DKSH Vietnam, Damco Vietnam, etc. To select a representative sample, this current study chose the four biggest LDCs, viz., Gemadept, JBS Logistics, Vinalines Logistics, and Saigon Newport for the empirical case. After that, the research team asked these LDCs to provide 10 major customers. As a result, we had a sample of 40 respondents for the survey. Because of adopting BWM to determine CRAs' weight, referring to Rezaei (2015), this paper designed the nine-point pairwise-comparison scale, as presented in Table 3, to survey 40 respondents, as mentioned above. Note that due to the highly professional nature of the questions in the questionnaire, all of the participants being surveyed were required to have adequate professional expertise in the logistics industry. From April to July 2023, we managed to gather information from such 40 experts. Nevertheless, 3 responses were discarded as they did not satisfy the consistency of BWM. Consequently, only 37 responses are valid for the next analysis.

By the way, please refer to **Rows 234-248** for more information.

b) The experts and their background:

The experts in our research are customers of logistic distribution centers (LDCs). Besides, anwering questions in BWM is highly professional. Accordingly, we randomly sampled the most appropriate interviewees with the following prerequisite conditions: (1) at least 5 years of working

experience in LDCs, (2) holding the position of senior staff or above, and (3) over twenty-five years of age.

Respondents' background is also shown in Table 4. Evidently, 62.2% of respondents hold managerial positions at their workplaces while 56.8% of them have working experience of more than 11 years in the distribution field. Additionally, 70.3% of respondents working at LDCs have an operating year of more than 15 years. Moreover, some main types of products of LDCs include pharmaceuticals and healthcare products, industrial and construction equipment, Fast-Moving Consumer Goods (FMCG), apparel and fashion, automotive parts and accessories, and electronics and telecommunications equipment.

By the way, please refer to **Rows 251-256 and Rows 620-621** for more information.

Once again, thank you for your dedication to advancing scientific knowledge and for your commitment to maintaining the high standards of PLOS ONE. We greatly appreciate your support throughout this process. If you have any further questions or require additional information, please do not hesitate to contact us.

Sincerely,

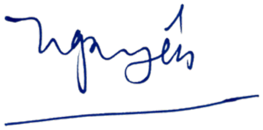

Nguyen Tan Huynh

huynhtannguyen@dntu.edu.vn

Faculty of Economics-Management, Dong Nai Technology University, Bien Hoa City, Vietnam.
