# Supplementary material for: The performance model of logistic distribution centers: Quality function deployment based on the Best-Worst Method
Source: PLoS One. 2024 Oct 31;19(10):e0305202. doi: 10.1371/journal.pone.0305202 (PMC11527329; doi:10.1371/journal.pone.0305202)
Supplement: Attachment — Submitted filename: Comments on PONE-D-23-31891.docx [file pone.0305202.s002.docx]

The authors of this paper conducted a study on the performance evaluation of LDCs by integrating the BWM and QFD methods. The overall structure and content of this work are acceptable. However, the following suggestions are provided for the authors to consider when improving the quality of your work:

1. The research background of the paper does not clearly explain why the QFD and BWM methods are necessary and how they are relevant to the research problem. Where do these methods align with the study?

2. The literature review section needs to be rewritten. Currently, it is simply a list without explaining the connections between the literature and why these specific ones were chosen. As we all know there is a vast amount of research on the performance evaluation of LDCs, so we recommend the authors present and compare the literature in a table format. Additionally, we did not see any analysis of the existing literature, particularly the shortcomings of previous studies and how this paper intends to address them.

3. What is the logical framework of the CRA system constructed in this paper? It appears that the authors used the SERVQUAL model, but what is the relationship or difference between it and the existing evaluation indicators?

4. The methods used in the paper are mature methods proposed by others, and we are concerned about the originality or novelty of the paper itself. The authors should summarize and emphasize the innovative aspects of this study.

5. The paper does not provide a clear description of how to transition from CRAs to STRs. It seems that STRs were derived based on expert opinions without utilizing the HOQ tool.

6. The case in the paper involves a multi-person collaborative decision-making problem. We recommend the author to use group decision-making methods, especially considering the influence of different experts' weights on the decision outcome.

7. The weights of the indicators have a significant impact on the final ranking of LDCs. The paper uses subjective evaluation data for this, so to enhance the robustness of the research, we suggest the author perform sensitivity analysis on the indicator weights. The following papers related to QFD and MCDMs may provide insights into this issue and should be referenced. (a) A Hesitant Fuzzy Linguistic QFD Approach for Formulating Sustainable Supplier Development Programs doi:10.1016/j.ijpe.2022.108428; (b) Large-scale group decision-making for prioritizing engineering characteristics in quality function deployment under comparative linguistic environment. doi:10.1016/j.asoc.2022.109359, (c) Enhancing Quality Function Deployment Through the Integration of Rough Set and Ordinal Priority Approach: A Case Study in Electric Vehicle Manufacturing etc.

8. The paper lacks a comparison with other quantitative research methods, which prevents it from demonstrating the superiority of this study. The case study only proves the feasibility of the research methods used in this paper.

9. The paper contains numerous grammar and spelling mistakes. For example, the LCD in section 2.2 should be LDC and the MWM in section 3.1 should be BWM. We recommend the author have a native English speaker carefully review and improve the writing.
